# Supplementary material for: Consistent Biomarkers and Related Pathogenesis Underlying Asthma Revealed by Systems Biology Approach
Source: Int J Mol Sci. 2019 Aug 19;20(16):4037. doi: 10.3390/ijms20164037 (PMC6720652; doi:10.3390/ijms20164037)
Supplement: Supplementary file 1 [file ijms-20-04037-s001.zip › ijms-566768-supplementary-XML/Supplementary Material S2-Reports of Quality control for ten datasets.zip!/Supplementary Material S2-Reports of Quality control for ten datasets/QC_report_for_GSE44037/ma.pdf]

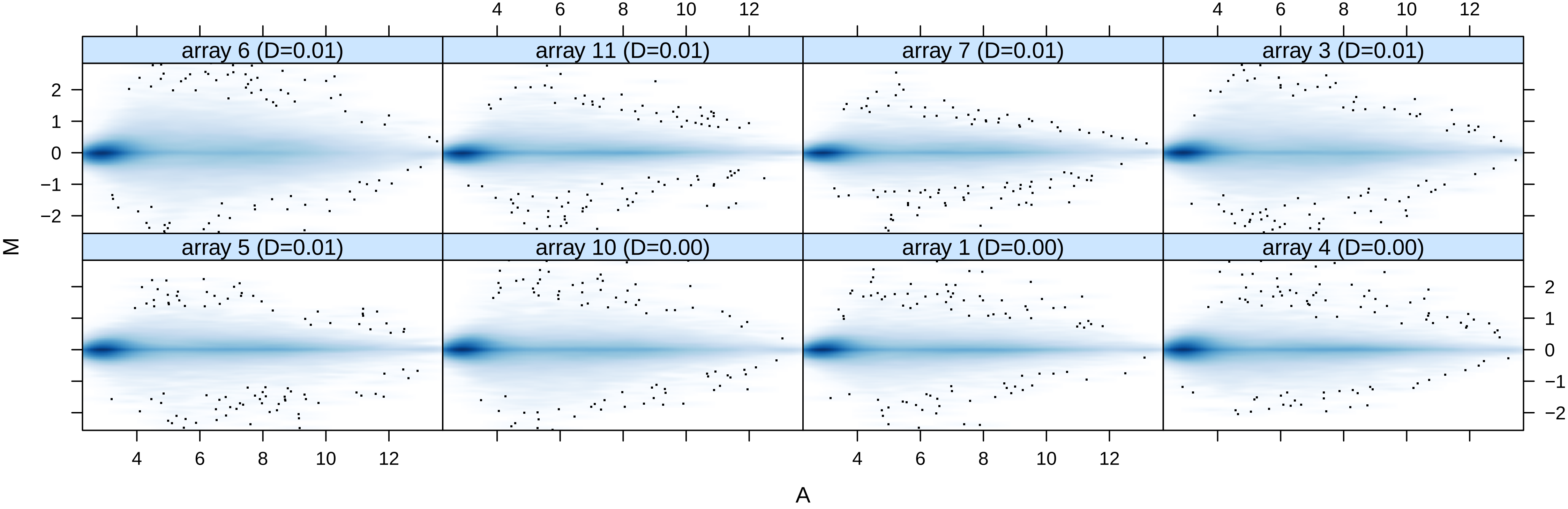

array 6 (D=0.01)
array 11 (D=0.01)
array 7 (D=0.01)
array 3 (D=0.01)
array 5 (D=0.01)
array 10 (D=0.00)
array 1 (D=0.00)
array 4 (D=0.00)
M
A
